# Potential Plasticity of the Mannoprotein Repertoire Associated to *Mycobacterium tuberculosis* Virulence Unveiled by Mass Spectrometry-Based Glycoproteomics

**DOI:** 10.3390/molecules25102348

**Published:** 2020-05-18

**Authors:** Laure Tonini, Bashir Sadet, Alexandre Stella, David Bouyssié, Jérôme Nigou, Odile Burlet-Schiltz, Michel Rivière

**Affiliations:** Institut de Pharmacologie et de Biologie Structurale, IPBS, Université de Toulouse, CNRS, UPS, 31077 Toulouse, France; laure.tonini@hotmail.fr (L.T.); bashir.sadet@gmail.com (B.S.); alexandre.stella@ipbs.fr (A.S.); david.bouyssie@ipbs.fr (D.B.); jerome.nigou@ipbs.fr (J.N.)

**Keywords:** glycoproteins, mannoproteins, mycobacterium, tuberculosis, protein-o-mannosyl transferase, mass-spectrometry, glycoproteomic

## Abstract

To date, *Mycobacterium tuberculosis* (Mtb) remains the world’s greatest infectious killer. The rise of multidrug-resistant strains stresses the need to identify new therapeutic targets to fight the epidemic. We previously demonstrated that bacterial protein-*O-*mannosylation is crucial for Mtb infectiousness, renewing the interest of the bacterial-secreted mannoproteins as potential drug-targetable virulence factors. The difficulty of inventorying the mannoprotein repertoire expressed by Mtb led us to design a stringent multi-step workflow for the reliable identification of glycosylated peptides by large-scale mass spectrometry-based proteomics. Applied to the differential analyses of glycoproteins secreted by the wild-type Mtb strain—and by its derived mutant invalidated for the protein-*O-*mannosylating enzyme PMTub—this approach led to the identification of not only most already known mannoproteins, but also of yet-unknown mannosylated proteins. In addition, analysis of the glycoproteome expressed by the isogenic recombinant Mtb strain overexpressing the PMTub gene revealed an unexpected mannosylation of proteins, with predicted or demonstrated functions in Mtb growth and interaction with the host cell. Since in parallel, a transient increased expression of the PMTub gene has been observed in the wild-type bacilli when infecting macrophages, our results strongly suggest that the Mtb mannoproteome may undergo adaptive regulation during infection of the host cells. Overall, our results provide deeper insights into the complexity of the repertoire of mannosylated proteins expressed by Mtb, and open the way to novel opportunities to search for still-unexploited potential therapeutic targets.

## 1. Introduction

Although curable, human pulmonary tuberculosis (TB) caused by *Mycobacterium, tuberculosis* (Mtb) remains the deadliest bacterial infection, with more than one death every 30 s [1]. Today, the disease burden is worsened by the increasing incidence of co-infection by HIV and the threatening emergence of multi- or extensively-drug-resistant Mtb strains. To face this alarming situation, efforts must intensify to foster the development of innovative protective and therapeutic strategies to fight TB [2]. Among these, the search of so-called “anti-virulence drugs” able to disarm the pathogen of its virulence without affecting its vital metabolic pathways is considered as a promising approach to circumvent the emergence of antibio-resistance [3]. In this context, we recently demonstrated that secreted proteins which are *O-*mannosylated by the Mtb protein-*O-*mannosyl transferase PMTub may constitute a new class of virulence factors dispensable for Mtb growth in vitro but essential for its infectiousness and virulence in a rodent animal model of TB [4]. However, among the very few Mtb *O-*mannosylated proteins formally characterized to date [5,6,7,8,9,10,11,12,13,14,15,16], none can account by itself for the pathogenicity. Thereby, the link between protein-*O-*mannosylation and the virulence of Mtb remains still poorly understood.

Thus, the comprehensive exploration of the repertoire of *O-*mannoproteins secreted by Mtb and the molecular characterization of their glycosylation pattern constitute key steps for a deeper understanding of the functional impact of the mannosylation on the biologic activity of the mannoproteins associated to Mtb virulence.

Mass spectrometry (MS)-based proteomics represents a powerful strategy to study posttranslational modifications of proteins. However, the large-scale characterization of glycoproteome still remains highly challenging [17,18,19,20,21,22,23,24,25] even in the case of glycosylation motifs of limited structural diversity such as the Mtb mannoprotein appendages which consist in one to four linearly linked mannose units substituting Ser or Thr residues [8,9,26].

To our knowledge, only few representative large-scale studies have tried to address the complexity of the Mtb mannoproteome to date. Among these, using an unbiased approach combining SDS page protein fractionation and MS-based proteomics, Birhanu et al. [27] reported very recently, an extensive inventory of the Mtb cellular glycoproteome. This latter encompasses all forms of glycoproteins regardless of their glycosylation type (Asn-*N*- or Ser/Thr-*O-*glycosylation), their glycan moiety (heptose, hexose, deoxyhexose, pentose, *N*-Acetylated hexose, etc.) or the nature of the sugar mostly found in mycobacterial glycoconjugates (MurA, Glc, Gal, Man, Rha, Fuc, Ara, etc.) [27]. In this study, 2944 glycopeptides were detected that derive from 1325 potentially glycosylated proteins corresponding to almost 1/3 of the Mtb gene-coded proteins. Interestingly, 294 of these glycopeptides correspond to Ser- or Thr-*O-*substituted peptides bearing single hexose moieties or short hexose oligomers (two to three units). However, the nature of the hexose stereoisomers substituting the peptides (Man, Glc, Gal, for the most commonly reported) cannot be deduced from the mass spectrometry data because of their isobaric masse. Therefore, this study does not permit to determine accurately, which of the 173 glycoproteins matching to these *O-*glycosylated peptides, are actually mannoproteins.

At the opposite, Gonzales-Zamorano et al. [10] used a targeted approach based on a specific Con-A affinity chromatography step to enrich selectively the mannosylated protein fraction. This strategy coupled to 2D gel-electrophoresis and MS-based proteomics allowed the identification of 41 potentially mannosylated proteins in a culture filtrate (CF) of Mtb. However, the approach developed in this landmark work did not provide any glycopeptide identification that could confirm the formal glycosylation of the proteins nor afford structural clues that could help to unveil the biologic function of the mannosylation.

Alternatively, Smith et al. [12] applied a similar pre-analytical lectin-affinity enrichment procedure at the peptidic level for the large-scale *O-*linked glycosylation sites profiling of Mtb culture filtrate proteins. This approach, combined with LC–MS/MS analysis of the enriched glycopeptide fraction, led to the characterization of 14 glycosylated peptides mapping 13 mannosylated proteins in a protein digest of a Mtb CF. However, of the five *O-*mannosylated proteins formally characterized until then in Mtb, four were not detected in this study, i.e., LpqH [6], Mpt83 [8], SodC [9] and LprF [11]. One of the reasons that could explain the absence of these emblematic Mtb mannoproteins is the limited exhaustiveness of the lectin affinity purification approach that constitutes a serious restriction for the comprehensive definition of the Mtb mannoprotein repertoire [28,29].

Overall, despite these dedicated strategies, direct evidence of mannosylation in Mtb lacks for almost two—third of the 47 mycobacterial glycosylated proteins recently reviewed by Mehaffy et al [30]. Hence, in order to identify new potential virulence factors of Mtb and to provide structural clues on the structural bases underlying the functional roles of protein *O-*mannosylation, we undertook a methodical glycopeptidomic exploration of the specific PMTub associated mannoproteome. With this aim, we set up an unbiased glycoproteomic approach to identify the most probable mannoproteins associated to Mtb virulence by comparing the glycoproteomes expressed by the wild-type H37Rv Mtb strain (Mtb^WT^) and its derived MtbΔrv1002c mutant invalidated for the protein-*O-*mannosyl transferase PMTub.

We applied also this approach which combines SDS–PAGE fractionation and MS-based proteomics with a stringent multi-parametric filtering strategy for high confidence identification of the glycopeptides, to explore conversely the impact of the constitutive overexpression of the PMTub gene on the contour of the mannoprotein repertoire.

Owing to this, we were able to characterize more than one hundred glycopeptides that were not detected in the MtbΔrv1002c mutant and which can be attributed obviously to PMTub modified mannoproteins. Not all, but most of the glycopeptides from the known mannoproteins previously found in the Mtb CF were detected as described or with related sequences and/or different glycosylation pattern. More interestingly, beside these latter, most the selected hits, was found to correspond to new glycopeptides that fulfill a stringent validation procedure providing reliable structural evidence of glycosylation for about twenty five new Mtb proteins.

Finally, we tentatively addressed the consequences of the regulation of the PMTub gene expression on the variability of the mannoprotein repertoire expressed by Mtb during the infection. Our results will pave the way to a better understanding of the contribution of mannoproteins in Mtb virulence.

## 2. Results

### 2.1. Mtb Culture Filtrate Proteome Coverage Using SDS–PAGE Separation and MS-Based Shotgun Proteomics

Mtb mannosylated proteins are secreted and accumulate in the culture filtrate (CF) all along the growth of the bacteria. Hence, the MS-based proteomic analyses were performed on protein extracts from 6 weeks old Mtb cultures grown on a glycerol-based synthetic medium. These conditions being considered as the optimal ones to recover the highest amount of mannoproteins [7,8,9,10,12]. The complexity of CF protein mixtures was resolved by SDS–PAGE and the gel strip was sliced into 17 bands that were processed in parallel for in gel digestion and peptide extraction. The individual peptide extracts were successively analyzed by nano LC–MS/MS using collision induced dissociation (CID) as activation method. This fragmentation mode allows to maximize the MS^2^ sampling for optimal analytical coverage of the peptide mixture [31], but also permits to preserve the characteristic hexose neutral loss ions signing the peptide glycosylation [12,32,33]. The datasets of fragmentation spectra obtained for each of the 17 bands were individually processed for the data mining of the annotated Mtb TubercuList reference database (release 27; 4031 entries). The resulting search outputs were merged for protein validation using the in-house developed MFPaQ software [34].

Given as an example, the analyses of the 17 SDS–PAGE gel slices of the Mtb^WT^ culture filtrate (CF) fractionation resulted in the recording of 244,488 fragmentation spectra that were assigned to 13,602 unique peptide sequences mapping 1232 proteins identified with a protein false discovery rate of 1% (FDR 1%; *p* > 0.004) (Table S1, Figure S1). This protein repertoire covers about 31% of the total annotated gene-coded proteome and ~40% of the expressed proteome [35] amenable to discovery using MS-based proteomics [36]. Most of the proteins detected herein (*n* = 1007; >81%) were previously reported by Albrethsen et al. (1362 proteins, [37]) from label-free 2D-DIGE proteomic analyses of the Mtb extracellular proteome (Table S1). The results obtained from these independent studies reveal a remarkable consistency of the Mtb culture filtrate proteome composed of a thousand proteins. It is noteworthy that only a limited number (95/1007; <10%;) of the proteins identified are actually predicted to be secreted and hence susceptible to correspond to secreted mannoproteins [38].

Similar analyses of the secreted proteome of the Mtb PMTub mutant (ΔRv1002c) led to very comparable results with the identification of 1187 proteins (FDR < 1%) present in the culture filtrate (Table S2) including 935 (78%) proteins shared with the dataset of Albrethsen et al [37].

The substantial coverage of the Mtb secretome achieved, using this pre-analytic SDS–PAGE fractionation approach, supports its efficiency and relevance to maximize the detection of low-abundance glycosylated peptides as also reported by others [27]. Nevertheless, while suited for an in-depth qualitative proteome exploration, the analyses of the multiple fractions is also expected to lead to less accurate differential quantitative analyses than single fraction comparisons. Therefore, we performed a complementary quantitative analysis of the relative expression of the glycoproteins detected in the unfractionated extracts to verify whether it could be affected by the interruption of the PMTub catalyzed mannosylation. Label free quantification of the non-glycosylated prototypic peptides showed no statistically significant differences for each of the putative mannoproteins detected in the CFs of the Mtb^WT^ and the MtbΔrv1002c mutant (Figure S2). This result obviously suggests that the disruption of the protein-*O-*mannosylation process does not affect the expression of the target proteins.

### 2.2. Identification of Glycopeptides Present in the Culture Filtrate of Mtb

In an initial attempt to identify the glycopeptides deriving from the already known mannoproteins present in the Mtb^WT^ CF, we applied a targeted search strategy. Briefly, the Mascot data-mining engine was parametrized for the identification of the presence of mono-, di—and tri-hexosyl modifications substituting Ser or tThre as variable labile post-translational modifications (PTMs). Hence, according to the difference between the experimental and the calculated mass of the peptide inferred from the fragment ions series of the non-glycosylated peptide sequence observed in the CID MS^2^ spectrum, about 1291 glycopeptide were identified mapping 577 putative *O-*glycosylated proteins (Figure S1). Because of the absence of additional available screening criterion to verify individually each of these putative assignments, we focused first on the search for the mannosylated peptides previously reported in Mtb [7,8,9,11,12,39,40,41]. This targeted search led to the identification of 103 MS^2^ spectra assigned to glycopeptides matching with 11 of the 17 Mtb mannoproteins biochemically confirmed to date (Table 1).

### 2.3. Improvement of MS^2^ Data Processing for Mtb Glycoproteins Discovery

However, about 98.5% of the 7779 MS^2^ spectra attributed to glycosylated peptides remained speculative requiring further endorsement (Figure S1). This considerable number of spectra to process makes such a task hardly manageable manually. To overcome this hurdle, we set up an automated procedure to search for fragmentation spectra exhibiting the characteristic signature of glycosylation that could strengthen the reliability of the putative assignments for new glycopeptides discovery. Indeed, CID fragmentation spectra of glycoconjugates bearing terminal hexoses are characterized by the presence of intense Y-type fragment ions resulting from the loss of the sugar ring(s) as neutral fragments [12,32,41]. Most of the CID-MS^2^-data search engine algorithms can be parametrized to take advantage of this property for the recognition of glycopeptide fragmentation spectra. However, they generally fail to exploit the full discriminating value of these diagnostic ions by miscarrying their relative intensity or rank in the fragmentation spectra. To improve the selectivity of the glycopeptide identification, we thus computerized a screening procedure to detect the MS^2^ spectra exhibiting consistent glycosylation signature ions clearly emerging from the background noise. Applying this filtering strategy, we found that only a very minor proportion (*n* = 223) of the MS^2^ spectra assigned to putative glycopeptides actually exhibited the expected signature (Figure S1, Table S2). It is noteworthy that 30% (*n* = 67) of these fragmentation spectra corresponded to previously reported glycopeptides of 8 known Mtb mannoproteins while the remaining 156 “signed” spectra pointed to 120 previously unreported glycoprotein candidate.

### 2.4. High Confidence Identification of Mtb Glycosylated Proteins

Although highly stringent with less than 3% of validated MS^2^ spectra, it cannot be totally excluded that this filtering strategy still allows the endorsement of false positive glycopeptide assignments. Then to increase further the reliability of the glycopeptide identification, we added two additional selection parameters. First, based on the observation that a large majority (>74%) of these assigned spectra corresponds to single sequencing events (Table S2)—and considering the stochastic character of the precursor ion selection for fragmentation [36]—we deliberately chose to temporary set apart the “orphan” spectra and to consider exclusively the assignments relying on several matching spectra. This resulted in the subsequent validation of a very limited number of precursor ions spectra (*n* = 40) that identify 10 peptides potentially glycosylated (Table 2).

**Figure 1:**
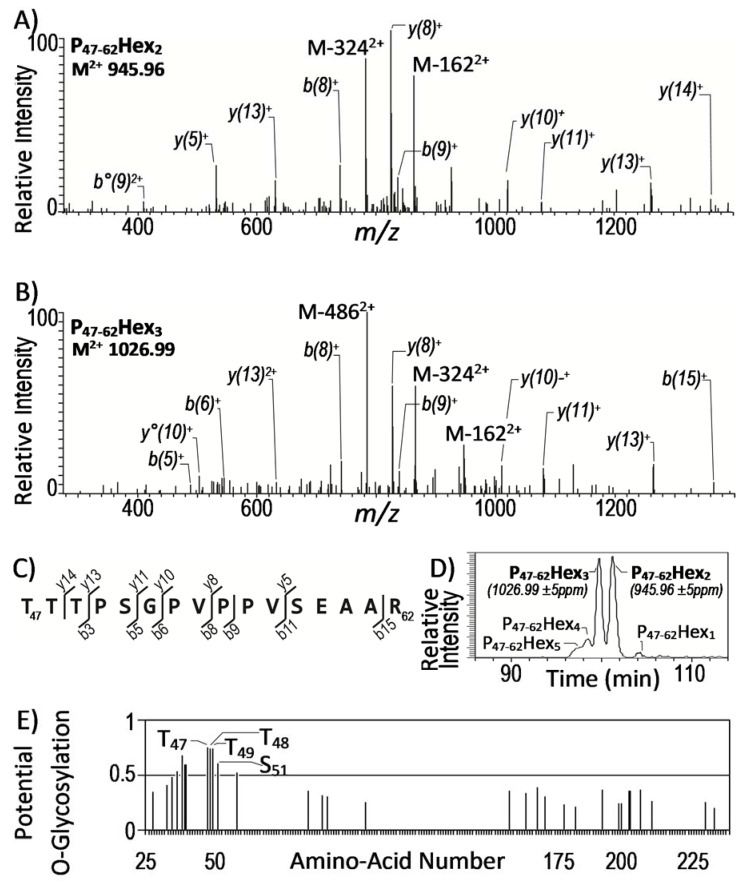
Description of the multi-parametric confirmation of the glycosylation of peptide T47-R62 of the lipoprotein LpqR (UniProtKB Access. # O53850; Mtb gene Rv0838) as an illustration of the approach used for high confidence identification of new Mtb glycopeptides from the LC–MS/MS (CID) data. (**A**) and (**B**) annotated CID mass spectra of the doubly charged precursor ions (M + 2H)^2+^ at 945.96 Da and 1026.99 Da displaying respectively the expected characteristic signature fragment ions M − 1622^+^, M − 3242^+^ or M − 1622^+^, M − 3242^+^ and M − 4862^+^ corresponding to the neutral loss of 1 to 3 hexose residues and supporting the identification of the di-substituted and tri-substituted glycoforms of the tryptic peptide T47-R62 of the LpqR lipoprotein; (**C**) sequence of the LpqR tryptic peptide T47-R62 reporting the peptide sequence fragment ions observed in the MS^2^; (**D**) extracted ion chromatogram (with of an ion mass tolerance of ±5 ppm) of the different glycoforms detected for the LpqR peptide T47-R62 validating the coherence of the elution time as inversely related to the glycosylation degree of the peptide; (**E**) NetOGlyc 4.0 predictions of the *O-*glycosylation sites of the LpqR sequence assessing the highest glycosylation probability of the Threonine triad T47-T49 and of the Serine S51 of the peptide T47-R62 (NetOGlyc: [42]). Additional data confirming the glycosylation of the Rv0315 and Rv1411c proteins are available in Supplementary Figure S3.

Second, still to gain more confidence into the assignment of these spectra, we took advantage of the well-established intrinsic micro-heterogeneity of the glycosylation process to select the most probable glycopeptide candidates. Indeed, alike most glycosylation routes, the protein glycosyl post-translational modification leads to the formation of structurally related glycoforms differing by their carbohydrate content (number and/or nature of the sugar). Accordingly, among the 13 precursor ions (Table 2), two of them, i.e., M = 1889.9 ([M + 2H]^2+^ = 945.96) and M = 2051.9 ([M + 2H]^2+^ = 1026.99) showed a mass difference of 162 amu that readily allows their assignment to different glycosylation states of the LpqR P47–62 peptide bearing 2 and 3 hexoses, respectively (Figure 1a,b). These assignments were further supported by the analysis of the respective relative LC–MS elution time of these glycoforms. Indeed, the lower chromatographic retention time of the expected most glycosylated form at M = 2051.9 is consistent with its higher polarity (Figure 1d). The consistency of the chromatographic behavior of these glycosyl-modified peptides [42] together with the presence in the sequence of a Ser or Thr rich cluster (Figure 1c) with a high glycosylation probability (Figure 1e), permit us to propose the LpqR (Rv0838) as a novel glycosylated protein of Mtb.

In contrast, the identification of the remaining 8 glycopeptide candidates seems less reliable due to the lack similar glycoforms. However, the assignments of 3 of these precursor ion masses at M = 3615.7, M = 3544.7 and M = 1115.6 are supported in a same way, by the presence of the related lower mass ions at M = 3453.68, M = 3382.64 and M = 953.55. Indeed, compared to the former ones, these latter present a mass difference of 162 amu that is readily attributable to the absence of an hexose residue. Moreover, they exhibit fragmentation spectra and chromatographic behaviors consistent with the non-glycosylated forms of the corresponding peptides (Figure S3). Thus, the concomitant presence of both the non-modified tryptic peptides and their mono-glycosylated forms corroborated their respective assignments and was considered as above, as a consistent proof of glycosylation of the hypothetical proteins Rv0315 and of the virulence associated lipoglycans transporter LprG.

Finally, the failure to detect the unsubstituted peptide or any related glycoforms for the 5 remaining precursor ion masses M = 1658.7, M = 1632.8, M = 1497.7, M = 1705.8 and M = 3813.8 in Table 2, makes these assignments less reliable even though fulfilling most of the usually accepted criteria for glycopeptide characterization. Therefore, although their glycosylation cannot be strictly ruled out in absence of definite indications, these candidates were not considered in this study.

### 2.5. Assessment of the PMTub Associated Mannoprotein Repertoire

To verify whether the newly identified glycopeptides correspond to the PMTub associated mannoprotein repertoire we analyzed in parallel a CF protein extract from the Mtb Δrv1002c mutant invalidated for the protein *O-*mannosyl transferase gene using the same multi-parametric selection workflow. In these conditions, we found 138 spectra assigned to putative glycosylated peptides that exhibit the neutral loss signature (Table S3). Targeted search on the full MS^2^ dataset failed to detect any fragmentation spectra fitting with known glycopeptides. Moreover, none of these spectra corresponded to any of the expected glycopeptides of the newly Mtb mannosylated proteins identified above. Finally, after the exclusion of the MS^2^ spectra corresponding to unique sequencing events (123/138), only 15 fragmentation spectra attributed to 4 putative glycopeptides remained. However, manual curation of these latter candidates failed to identify associated glycoforms that would confirm, as above, the glycosylation of these peptides. It is noteworthy that, the absence of reliable identification of putative glycopeptides in the Mtb Δrv1002c mutant suggests that the secreted hexosyl modified proteins present in the Mtb culture filtrate are exclusively PMTub modified mannoproteins.

In conclusion, using this original, strategy 42 glycopeptides detected in the culture filtrate of the Mtb^WT,^ but not in the Mtb Δrv1002c mutant one, could be assigned with high-confidence to Mtb mannoproteins. Of these, 37 obviously match with characterized glycosylated peptides from previously described Mtb mannoproteins, while 5 correspond to novel glycopeptides belonging to 3 proteins: the DC maturation-inducing antigen Rv0315, the Mtb virulence-associated Rv1411c LprG lipoprotein and the Rv0838 D-alanyl-D-alanine dipeptidase lipoglycoprotein LpqR (Table 1 and Table 2). It is worth mentioning that while Rv0315 and LprG were both presumed to be mannosylated [10,40,43,44,45], our data provide the first convincing evidence of mannosylation of the Rv0838 LpqR C-terminal peptide.

### 2.6. Variability of the Mtb Mannoproteome Determination

**Figure 2:**
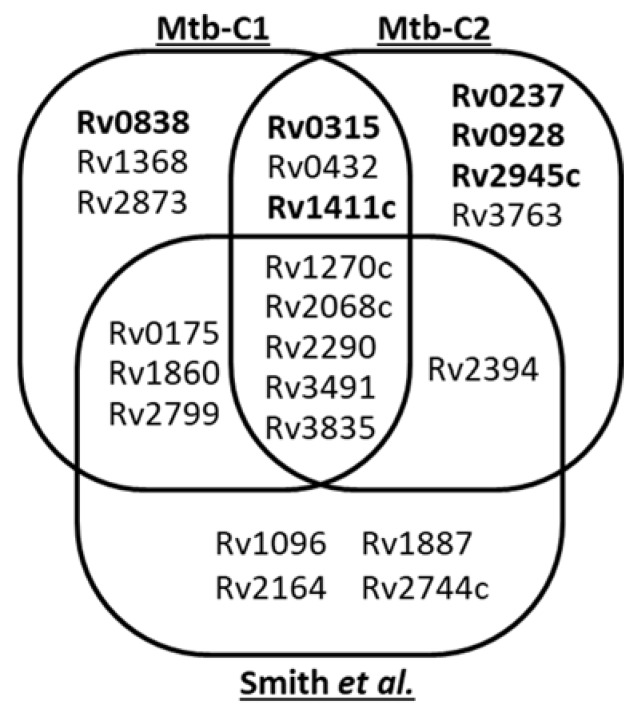
Variability of the Mtb H37Rv secreted glyco-proteome determination based on glycopeptide characterization: Venn diagram comparing the sets of putative mannoproteins identified with our SDS–PAGE based MS shotgun glycoproteomic approach (duplicate analyses Mtb-C1 and Mtb-C2) and with the pre-analytical lectin based glycopeptide enrichment used by Smith et al. These different analyses identify a total of 23 putative Mtb glycoproteins, but with a rather limited core of 5 glycoproteins shared by the 3 samples and a similar proportion of sample specific candidates (between 3 and 4). 2 by 2 analyses show very comparable results and do not evidence major quantitative divergences between the two analytical approaches used. (Novel glycoproteins are noted in bold).

It is striking that only 8 of the mannoproteins detected herein were also reported by Smith et al. [12]. Indeed, we did not detected the glycopeptides reported by these authors for the Rv1096, Rv1887, Rv2164c, Rv2394 and Rv2744c proteins (Figure 2). It is clear that the original filtering strategy we used as well as the culture conditions and the origin of the Mtb strain analyzed could affect the output of our analyses and explain these differences. To verify this assumption, we duplicated our analysis using strictly the same Mtb culture conditions, pre-analytical treatment and data processing. Examination of the CF proteins of this second replicate led to a very comparable number of hits with 13 mannoproteins identified (Table 3). However, the nature of the mannoproteins identified substantially diverge qualitatively with, respectively, 62% (8/13) and 42% (6/13) overlapping with our first biologic replicate or with the Smith et al. inventory (Figure 2). Precisely, on one hand, proofs of glycosylation are missing for the conserved Mce associated membrane protein Rv0175 [12], LprF (Rv1368, [11]), Apa (Rv1860, [7]), Rv2799 [12], as well as for the LpqR (Rv0838) and the antigenic MPB83 (Rv2873, [8]), which were not detected at all in this second sample. On the other hand, compared with our initial analysis, 5 additional glycopeptides were detected including 3 that were readily assigned to the previously reported glycopeptides P27–46 of the γ-glutamyl transferase GgtB (Rv2394) [12], P39–55 of the LppX (Rv2945; [46]) and P27–51 of the 19kDa antigen LpqH (Rv3763; [41]). Interestingly the two remaining hits correspond to novel glycosylated peptides supporting the presumed mannosylation of the two proteins LpqI (Rv0237) and PstS3 (Rv0928) [10] (Figure S4).

Finally, of the 23 glycoproteins identified in total by Smith et al. [12] and us, only 5 (<22%) were systematically detected in the 3 analyses while 12 (52%) were present in only 2 experiments and 11 (48%) were sample specific (detected in a single essay).

Although these differences are generally attributed in large part to the technical variability arising from the intrinsic stochastic character of the selection of low abundance parent ions for MS^2^ analysis [47], other factors, such as the most likely inherent biologic variability of the glycoproteome expressed by the bacteria from culture to culture may contribute also to these discrepancies. Indeed, even under highly defined and controlled cultivation conditions, subtle environmental ill-defined variations or fluctuations of poorly understood cellular processes, are known to provoke significant qualitative and quantitative alterations of the protein expression and post-translational modification [48,49]. Hence, one can reasonably speculates that the observed disparity of the Mtb mannoprotein repertoires identified from sample to sample results from a stochastic behavior of the protein-*O-*mannosylation process that generates an intrinsic diversity of the mannoproteins secreted as recently reported for the S-palmitoylation of membrane proteins [50].

### 2.7. Exploration of the Mtb Cell Associated Glycoproteome

**Figure 3:**
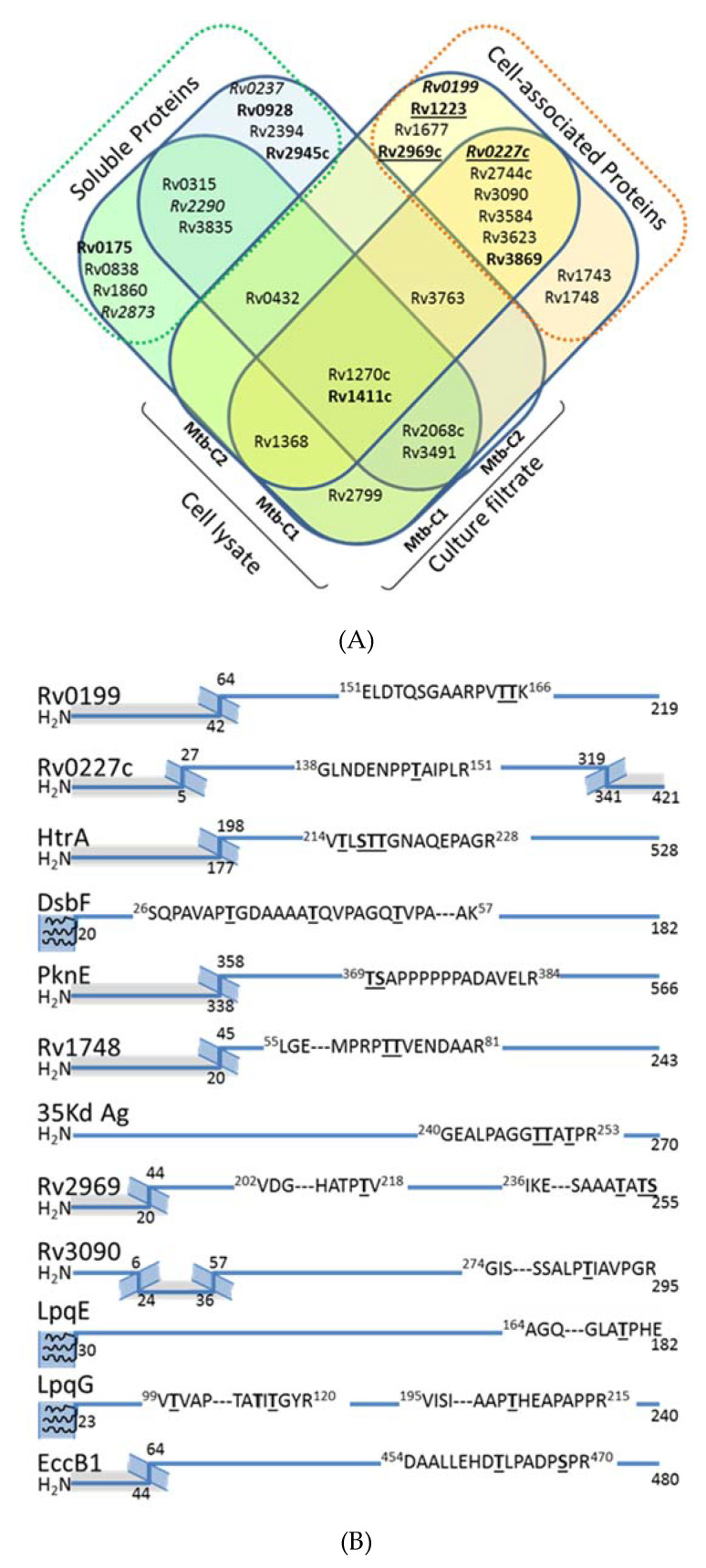
Analysis of the cell-associated glycoproteome of Mtb^WT^ reveals the presence of envelope specific glycosylated proteins. (**A**) Venn diagram comparing the mannoprotein repertoire identified from the culture filtrate and cell–lysate protein extracts from two independent culture Mtb^WT^ (Mtb-C1; Mtb-C2). Hypothetical proteins are noted in italic, while essential proteins for in vivo and in vitro are in bold and bold-underlined, respectively; (**B**) Topographical analysis of the putative glycosylated peptide sequences of the newly identified envelope associated glycoproteins of Mtb^WT^ localizes the glycosylated motif systematically in an extracellular segment. (Protein sequences are symbolized by blue lines; gray zones correspond to putative intracellular domains according TMHMM 2.0; the membrane spanning peptides are represented by hatched motifs; putative lipoproteins are represented with 3 terminal membrane anchored acyl groups figured by curved lines; the putative *O-*glycosylation sites with the highest occupancy probability according to NetOGlyc 4.0 predictions are indicated by underscored Ser or Thr residues).

Alongside these analyses, we also examined the presence of potential mannoproteins in the Mtb cell lysate. Indeed, since the seminal work of C. Espitia & R. Mancilla [5] and in agreement with the current model of the mycobacterial protein glycosylation pathway [51], all the studies dedicated to the identification and characterization of mycobacterial mannoproteins have focused exclusively on the soluble proteins present in the CF. However, it is noteworthy that many of the *bona fide* or suspected Mtb glycoproteins reported to date are also putative lipoproteins, which may remain more tightly associated to the bacterial cell wall through their lipidic anchor [41]. Therefore, we explored the cell associated glycoprotein repertoire from protein extracts obtained from the disrupted bacterial cells of the two Mtb biologic replicates. In total, the overall glycopeptidomics analyses of these fractions afforded high-confidence indications of glycosylation for 44 peptidic sequences attributed to the different glycoforms of 25 putative glycopeptides mapping 20 glycoproteins (Table 4). Of these, only 8 correspond to glycoproteins also detected in CFs of Mtb which were previously annotated as either putative lipoproteins or secreted proteins (Figure 3A). In contrast, the remaining 12 glycosylated proteins identified are actually novel Mtb mannoproteins considered from literature as intimately associated to the cell envelope [52]. However, unexpectedly only 4 of them correspond to lipoproteins or peripheral membrane proteins as initially hypothesized: i.e., the extracellular disulfide bond forming protein Dsbf (Rv1677), the LpqE (Rv3584) and LpqG (Rv3623) lipoproteins and the phage shock protein orthologue PspA (Rv2744c)). Indeed, most these mannosylated proteins are classified as integral polytopic membrane proteins that have never been considered, up to now, as potential targets for glycosylation. However, it is worth mentioning that the topological analysis of the sequences of these eight proteins revealed that the glycosylated peptides correspond systematically to predicted extracellular domains flanked by hydrophobic transmembrane helices (Figure 3B). This observation is consistent with the expected membrane location of the protein glycosylation machinery and strongly supports a glycosylation process occurring co-translationally during the protein export /insertion in the membrane via the Sec dependent protein secretion system.

Finally, it is noteworthy that, alike the CF, the comparison of the cell associated protein extracts, revealed significant differences between the 2 biologic replicates with only half of the proteins shared between these two samples (10 of the 20 proteins identified).

### 2.8. Potential Impact of the Mtb PMT Expression on the Mtb Glycoprotein Repertoire

Despite the critical impact of the Mtb protein-*O-*mannosylation on the invasiveness and virulence of the infectious pathogen, the regulation of this process remains unexplored. Moreover, the recent report of the PMTub higher expression in the virulent W. Beijing strain than in lower virulence Mtb strains [53], further addresses the question of the correlation between the PMTub expression level and the contour of the repertoire of mannoproteins secreted by Mtb.

**Figure 4:**
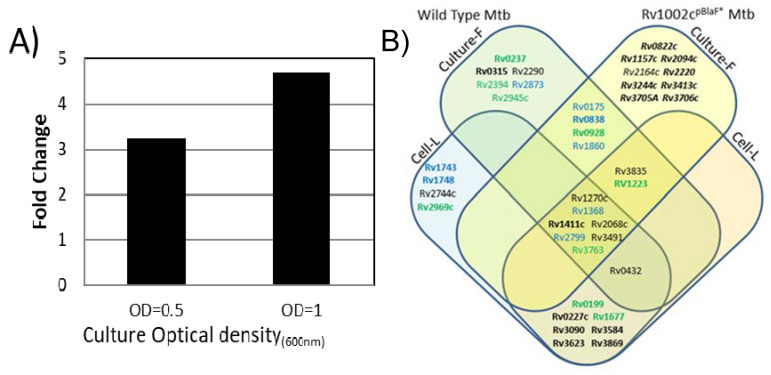
Influence of the PMTub increased expression on the mannoprotein repertoire expressed by Mtb. (**A**): constitutive higher expression level of the PMTub Rv1002c gene in vitro in the complemented ΔRv1002c: pBlaF*Rv1002c strain relative to that of the Mtb^WT^ strain during the exponential (DO = 0.5) and the stationary (DO = 1) growth phases (see Table S4 for raw data). (**B**) Repertoire of glycoproteins identified in the secretome of the PMTub-overexpressing strain under the control of the pBlaF* promotor. Venn diagram showing the set of mannoproteins shared between the Mtb complemented strain expressing the PMTub gene (Cp) and the two independent analyses of the Mtb^WT^ strain secretome (Mtb-C1 (blue); Mtb-C2 (green)). (Novel glycoproteins are noted in bold while complemented strain specific proteins are in italic; Culture-F: culture filtrate; Cell-L: cell lysate).

Therefore, we investigated whether and to what extent the Mtb mannoprotein repertoire was affected by an increased expression of PMTub. With the aim to evaluate exclusively the influence of a PMTub overexpression on the Mtb glycoproteome, independently of the genetic backgrounds of the Beijing Mtb lineage, we used an isogenic Mtb complemented strain expressing the PMTub gene under the control of a pBlaF* promoter [4]. Indeed, originally selected as a strong mycobacterial promoter, pBlaF* is regularly used for a high constitutive expression of the downstream gene [54]. This property was readily confirmed by quantitative PCR analysis that shows a 2 to 3-fold increase of the PMTub gene expression during both the exponential and stationary growth phases of the complemented strain compared to the Mtb^WT^ (Figure 4A).

The mannoproteome of the complemented strain was thus explored by SDS–PAGE fractionation and MS-based proteomics using strictly identical experimental settings to those used above for the parent Mtb^WT^. Interestingly, in these conditions, the number of proteins identified in the CF of the complemented strain was found very comparable to the one obtained with the Mtb^WT^ (1223 versus 1232 proteins). This result confirmed that the overexpression of the PMTub gene does not affect significantly, in its diversity, the repertoire of the secreted proteins expressed by the complemented Mtb strain (Table S5). In addition, we verified, by complementary label-free quantification experiments on the unfractionated extract, whether the expression of the known PMTub target proteins was altered by the overexpression of the PMTub gene. Again, quantification of the non-glycosylated prototypic peptides of the detected mannoproteins confirmed the absence of statistically significant differences in the relative abundances of the PMTub target protein between the parent Mtb^WT^ strain and the derived strain overexpressing the PMTub gene (Figure S5). In contrast, the glycopeptidomics analyses reveal that the overexpression of the PMTub gene is associated with a significant increase (+45%) in the number of different glycopeptides detected in the CF extract as compared to the parent Mtb^WT^ strain. Indeed, 61 different glycosylated peptides passing through the stringent filtering steps of our multi-parametric identification workflow were detected in the PMTub-overexpressing strain’s CF (Table S5). Most of them (43/61) match with glycopeptide sequences detected in the Mtb^WT^ by us or others (Figure 4B). But more interestingly, 23 of these glycopeptides that were never detected by us nor by others in the Mtb^WT^, were found to fit with 10 new peptidic sequences with variable glycosylation degrees. These new glycopeptides evidence definitely the unpredicted mannosylation of 8 additional Mtb proteins including some that are considered as essential such as the TatA Sec-independent protein translocase Rv2094c [55], the GlnA1 Glutamine synthase Rv2220 [55] and the MtrAB two-component system associated LpqB lipoprotein Rv3244c [56] (Table 5). Again, it cannot be totally excluded that the failure to detect these glycopeptides in the Mtb^WT^ is due to the stochastic character of the parent ion selection for fragmentation in MS/MS that discriminate the low abundance ions [47]. However, it is worth mentioning that these glycopeptides, which have never been reported to date in any of the previous Mtb^WT^ glycoproteome explorations, are identified here with high confidence by multipleMS^2^ scans. This result points to the PMTub-overexpression as the cause of the increase of the mannosylation of these proteins in the complemented strain up to a detectable level. Thereby, these results constitute strong evidences that the repertoire of the mannosylated proteins secreted by Mtb is affected in its diversity by the expression level of the PMTub.

### 2.9. Mtb PMTub Expression Is Increased in the Macrophage

**Figure 5:**
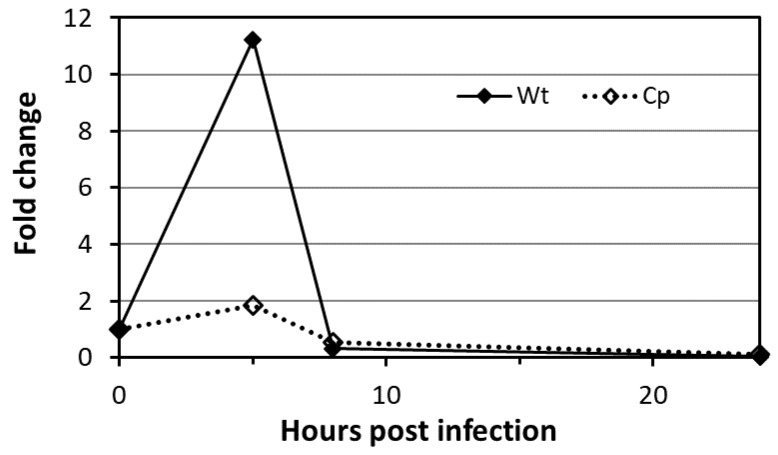
Regulation of the expression of the protein-*O-*mannosyl transferase PMTub (Rv1002c) gene in the Mtb^WT^ during the infection. Quantitative polymerase chain reaction (qPCR) measurement of the relative amount (compared to the housekeeping gene RpoB) of the Rv1002c amplicons in the Mtb^WT^ and in the complemented strain overexpressing constitutively the PMTub (Cp), during the infection of the murine alveolar macrophage cell line MH-S at a MOI of 10 (numerical data are available in Table S6).

Finally, to assess whether the Mtb mannoproteome may undergo modulation during the infection, we analyzed the expression of the PMTub gene in infected macrophages. Indeed, several evidences support a potential regulation of its expression in the host including. First, the immediate vicinity of rv1002c with the in vivo expressed iVEGI genomic island (running from rv0960 to rv1001) that has been shown to be specifically activated during mice infection [57]. Secondly, the presence of at least three binding sites for transcription regulatory factors upstream or within the rv1002c coding sequence reported by independent Mtb gene wide regulation network studies and suggesting a possible regulation of the gene transcription [58,59]. To support the physiological regulation of the PMTub gene expression in vivo, we thus monitored the expression of the Mtb rv1002c gene post-infection of murine alveolar macrophages. Interestingly, we observed that the host cell intracellular hostile environment induces a transient significant increased expression of the gene reaching its maximum 5 h post-infection (Figure 5).

In contrast, the expression of the PMTub gene was not affected when placed under the control of the constitutive pBlaF* promotor in the isogenic complemented mutant. These results confirmed the specificity of the promoter dependent transient upregulated expression of the PMTub gene during the colonization of the host cell. Whether this early increase of the PMTub gene expression in the macrophage could result in the mannosylation of subdominant glycoproteins specifically secreted during the infection and essential for the macrophage colonization and the intracellular survival of the virulent Mtb, remains to be demonstrated. Unfortunately, the evaluation of the impact of such increased expression of the PMTub gene on the Mtb mannoprotein repertoire expressed in vivo remains highly difficult to tackle due to the very low amount of bacterial proteins recoverable from infected macrophages.

## 3. Materials and Methods

Bacterial culture—*M. tuberculosis* (H37Rv) wild-type and RV1002c complemented ΔRv1002c mutant [4] strains were grown aerobically at 37 °C as pellicle for up to 6 weeks on glycerol-based Sauton medium for large scale glycoproteomic analyses. For macrophage infection purpose cells were routinely grown to exponential phase in Middlebrook 7H9 Media supplemented with ADC (Becton Dickinson Microbiology System) and 0.05% Tween−80 at 37 °C under shaking conditions.

Protein extracts—*M. tuberculosis* culture filtrate (CF) protein extracts were obtained after cell harvesting by twice filtration of the culture mediums on 0.22 µm membrane and concentration using Vivaspin 5k ultrafiltration devices (SartoriouStedim Biotech) of appropriated volume at the desire protein concentration for proteomics analysis. Harvested cell pellets in suspension (1/1; *v/v*) in lysis buffer (pH = 7.4; 50-mM Tris-HCl, 5-mM EDTA; 5-mM DTT, 1% SDS, Sigma P8340 Protease inhibitor cocktail) were disrupted by bead beating using 0.1-mm glass beads (3/1: *v/v*, MerckEurolab, France) at maximum speed for 30 s followed by 1 min cooling on ice (X 5 times). The homogenate was centrifuged at 12,000 g for 20 min at 4 °C. Pellet was discarded and the supernatant was recovered as the “Cell lysate protein extract”. The final protein concentration was estimated with the Bradford Protein Assay Kit (Thermo Scientific) according the manufacturer’s guidelines. Protein quality was checked by SDS–PAGE.

Gel Electrophoresis—Analytical and preparative gel electrophoresis for proteomics analysis were performed using, respectively 10-μg and 100-µg-equivalent protein / lane on 12% hand-cast analytical SDS-polyacrylamide gels (0.75- or 1.5-mm-thick, 8.3 × 7.3 cm) with a Mini-PROTEAN 3 electrophoresis system (Bio-Rad). Samples were run at a constant 20 mA for 1 h in 25-mM Tris, 192-mM glycine and 0.1% SDS and the gels were developed with InstantBlue (Expedeon) according manufacturer recommendation.

Mass spectrometry analysis—Gel lane was cut into 17 homogenous slices and treated as described [4]. The peptides mixtures were analyzed by nanoLC–MS/MS using an Ultimate3000 system (Dionex) coupled to an LTQ-Orbitrap Velos mass spectrometer (Thermo Fisher Scientific) operating in positive mode. The peptides were loaded on a 300-μm inner diameter × 5-mm PepMap C18 precolumn (LC Packings, Dionex) at 20 μL/min in 2% acetonitrile, 0.05% trifluoroacetic acid. After desalting for 5 min, peptides were separated on line on a 75-μm inner diameter × 15-cm C18 column (packed in-house with Reprosil C18-AQ Pur 3-μm resin, Dr. Maisch; Proxeon Biosystems, Odense, Denmark). Peptides were eluted using a 5%–50% gradient of solvent B during 80 min at 300 nL/min flow rate. The LTQ-Orbitrap was operated in data dependent acquisition mode with the XCalibur software. Survey scan MS were acquired in the Orbitrap on the 300–2000 m/z range with the resolution set to a value of 60,000. The twenty most intense ions per survey scan were selected for collision induced dissociation CID fragmentation and the resulting fragments were analyzed in the linear ion trap (LTQ). The normalized collision energy was set to 35% and activation times to 10 ms and 150 ms. Dynamic exclusion was employed within 30 s to limit repetitive selection of the same peptide. MS data are available via ProteomeXchange with identifier PXD012018 and the open source “Neutral-Loss finder” software is downloadable from https://github.com/david-bouyssie/neutral-loss-finder/. Post-analytical bioinformatics data processing

Database search and data validation—The Mascot Daemon software (version 2.6, Matrix Science, London) was used to perform database searches, using the Extract_msn.exe macro provided with Xcalibur (version 2.0 SR2, Thermo Fisher Scientific) to generate peak lists. The following parameters were set for creation of the peak lists: parent ions in the mass range 400–4500, no grouping of MS/MS scans, threshold at 1000. A peak list was created for each analyzed fraction (i.e., gel slice) and individual Mascot (version 2.6) searches were performed for each fraction. Data were searched against the TubercuList reference database (release 27; 4031 entries). The following list of variable modifications was used: carbamidomethylation of cysteines, propionamidation of cysteines, oxidation of methionine, mono-glycosylation (Hexose) of serine/threonine and di-glycosylation (2 Hexoses) of serine/threonine. Searches were performed using semi-tryptic digestion mode, and the specificity of trypsin was set for cleavage after K or R with two missed trypsin cleavage sites allowed. The mass tolerances in MS and MS/MS were set to 10 ppm and 0.6 Da, respectively, and the instrument setting was specified as “ESI-TRAP”. In order to calculate the False Discovery Rate (FDR), the search was performed using the “decoy” option in Mascot. Protein groups were validated based on this Mascot MudPIT score to obtain a FDR of 1% at the protein level: FDR = number of validated decoy hits / (number of validated target hits + number of validated decoy hits) x 100. The mass spectrometry proteomics data were deposited to the ProteomeXchange Consortium via the PRIDE [1] partner repository with the dataset identifier PXD012018

Filtering of glycosylated MS/MS spectra—To isolate MS/MS spectra resulting from the fragmentation of glycosylated peptides, peak lists (i.e., MGF files) were filtered using a dedicated algorithm searching for consecutive neutral losses of hexoses. The script written in the Perl language is executed on each MGF file. The algorithm starts by filtering the top N peaks (N = 30 by default, and minimum relative intensity > 10%) of a given MS/MS spectrum. Then, knowing the precursor ion m/z value and its charge (z), it computes the expected m/z value corresponding to the neutral loss of a given hexose: NLpeak = precursor_mz–(hexose_mass/z). A neutral loss is considered as being detected if the difference between the observed m/z value of the considered peak and the expected m/z value for this neutral loss is lower than a given tolerance (500 ppm in this study). If a peak is matched in the Top N list then the algorithm searches iteratively other consecutive neutral losses of the same hexose until no further loss is observed or until the maximum number of losses is reached (8 in this study). Finally, information about the putative glycosylated peptides are reported in a tabulated text file containing the precursor m/z and charge state, the spectrum title and the list of detected neutral losses. The neutral-loss finding software can be downloaded from https://github.com/david-bouyssie/neutral-loss-finder/.

Infections of macrophages—MH-S cell line macrophage infections were performed as previously described [4]. Briefly, macrophages in RPMI 1640 were infected with either the H37Rv wild-type strain or the rv1002c complemented Δrv1002c mutant at a multiplicity of infection (MOI) of 10. Infection was allowed to proceed at 37 °C for 1 h, and the extracellular bacteria were removed by 3 successive washes with fresh medium. At the end of the infection period, cells were harvested for RNA extraction at 0, 5, 8 and 24 h post-infection.

PCR amplification—Total cellular RNA was extracted from either in vitro cultured Mtb or infected MH-S macrophages with the RNeasy Total RNA kit, (Qiagen, Hilden, Germany) according manufacturer instruction. Reverse transcription was performed using the RevertAid retro transcription kit from Thermo Fisher. Quantitative PCR reaction was performed in 25-µL reactions using the Maxima SYBR Green kit and the following gene-specific primer pairs (sense and antisense) of the *M. tuberculosis* pmt rv1002c target gene and rpoB rv0667 housekeeping reference gene: Rv1002c-RTF: 5′ GTCTATCTGGCCACCTACGCT 3′, Rv1002c-RTR: 5′ GATTCCCAAGGGTGGTAGTTG 3′, Rv0667-RTF: 5′ AGGAACGGCATGTCCTCAAC 3′, Rv0667-RTR: 5′ CGAATCCGGCAAGGTGAT 3′. Reactions were performed on the Applied Biosystems 7500 and rounds of amplification and annealing temperatures were optimized for each primer pair. Analysis were performed using the manufacturer software

## 4. Discussion

From the pioneering works by Schultz et al. [60] and Espitia et al. [5], mycobacterial mannosylated proteins have aroused curiosity due to their potential contribution to the interactions between Mtb and its host [30]. However, the definitive evidence of their crucial role in the host-pathogen interaction only came with the demonstration of the essentiality of the protein-*O-*mannosylating enzyme for Mtb virulence [4]. Because protein *O-*mannosylation remains the sole post-translational protein glycosyl modification actually documented in Mtb [4,7,8,9,51], this finding renewed the interest for these potential virulence factors as a source for alternative chemotherapy targets [61] or new immuno-dominant epitopes for molecular vaccine development [62,63]. Although mannoproteins are of promising interest, the biologic functions of the protein glycan chains in Mtb still remain elusive [30,64]. Most of our knowledge is inferred from the studies dedicated to the immuno-dominant antigen Apa—an alanine and proline rich mannoprotein—specifically secreted by live bacilli [26,65,66,67]. Several Apa’s major biologic properties have been clearly ascribed to the presence of the mannosidic appendages. The mannosyl substituents are responsible for the binding of Apa to the host immune system C-Type lectines DC-Sign and SP-A, thereby, contributing directly to the invasion and the colonization of the host cell by the pathogen as reported since for several other mycobacterial mannoproteins [68,69]. In addition, changes in the mannosylation pattern of the *M. bovis* BCG Apa have been shown to alter its ability to stimulate CD4^+^ and CD8^+^ T-lymphocyte responses involved in the protective properties of the BCG vaccine against tuberculosis [70,71,72]. Alternatively, the *O-*mannosylation has been suggested to modulate the subcellular localization and the antigenic processing of the Mtb LpqH lipoprotein (19 kDa) antigen, [39,41,73]. In the case of the lipoglycan transporter lipoprotein LprG, the conclusions are less evident to draw since the glycan decorations were found dispensable for its transport function in Mtb [40] while they are essential for the MHC II restricted T Cell activation of lepromatous patient T lymphocytes [74].

However, in most cases, the role of the mannosyl decorations remains largely misunderstood due to the scarce number of precise structural characterization available but needed to determine with accuracy the structure-function relationships. A reason for this is that, while it is easy to evidence the presence of mannose residues on a protein using specific lectins, it is much more challenging to get clues on the localization of the glycosyl substituents on the protein skeleton. Such structural details are essential however to decipher the role of these appendages on the protein functionalities. Then to gain further structural insights into the *O-*mannosylation profiles of the Mtb proteins, we set up an unbiased strategy that combines a large-scale MS-based proteomic approach with the original search of multi-parametric signatures for the consistent characterization of glycopeptide from the LC–MS/MS data. The decisional pipeline combines (i) the detection in the MS^2^ spectra of fragment ions resulting from the neutral loss of hexose residues from the parent peptides, (ii) the presence of several MS^2^ spectra corresponding to different glycosylated forms of the peptides and (iii) the consistent chromatographic behavior of the glycoforms.

**Figure 6:**
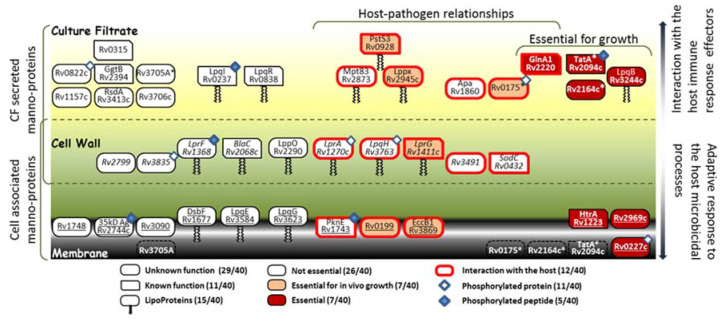
Topological distribution, essentiality and putative physiological and immunomodulatory properties of the Mtb mannoproteins identified. Mannoproteins exclusively detected in the CF and in the cell lysate (CL) extracts are depicted on the top and the bottom of the cartoon, respectively, while those detected in both fractions are represented in the intermediate space. The secreted mannoproteins detected in the CF must rather be involved in the interaction with the host immune response effectors while those more tightly associated to the envelope and detected in the CL must contribute to the bacterial fitness and to the adaptive response to the host microbicidal processes. The stars highlight the detection in the culture filtrate of glycopeptides arising from mannoproteins annotated as membrane proteins. Reported phosphorylated proteins are labeled with blue diamond and filled blue diamond when the identified glycopeptide matches with a phosphopeptide reported in literature.

Although we cannot rule out the improper exclusion of the less convincing spectra that did not meet one of these criteria (false negative spectra), this stringent filtering allowed nevertheless the narrow selection of about a hundred of the most reliable glycopeptide candidates present in the protein extracts of the Mtb^WT^ and the PMTub-overexpressing strains. Among the 40 Mtb mannoproteins that were identified with high confidence by their glycopeptides, 14 correspond to previously formally characterized mannoproteins, nine correspond to predicted one (from ConA binding experiment) and are structurally confirmed herein and 17 correspond to totally novel Mtb mannoproteins. Most of these (28 / 40) have no known function and are still considered as hypothetical or at the best as “probable proteins”. However, at least one third of these proteins are assumed to be essential for growth or/and for animal infection [75,76,77] (Figure 6). Here, we provide evidences of mannosylation for the first time for at least six of these “essential” proteins involved in the host pathogen-interaction: the Rv0227c (involved in the invasion and infection of Mtb target cells [78]), the HtrA-like serine protease Rv1223 [79], the Sec-independent protein translocase TatA (Rv2094c [80]), the Glutamine synthetase GlnA1 involved in the DCs maturation and activation (Rv2220; [81]) and the disulfide oxidase DsbA-like enzyme Rv2969c involved the formation of protein disulfide bonds essential for the protein folding [82]).

In addition, it is worth mentioning that among the glycosylated proteins identified some have been reported to be also phosphorylated on serine or threonine residues. Interestingly, comparison of our glycopeptide repertoire with the Mtb phosphopeptidomes reported in the literature [83,84,85,86] reveals for the first time that some peptides can be phosphorylated and glycosylated (Figure 6). The available data do not permit us to determine whether the two PTMs may be present simultaneously on the protein or are rather exclusive as in the case of the glycolipoprotein LpqH (Rv3763) [41]. Nevertheless, this finding addresses the intriguing question of the existence, in Mtb, of a functional interplay between protein mannosylation and phosphorylation analogous to the well described regulatory site-occupancy competition between phosphorylation and *O-*GlcNAcylation in the eukaryotes.

In addition, we found that the expression level of PMTub can alter the repertoire of mannoproteins expressed by Mtb. Indeed, in the strain overexpressing the PMTub gene, we have observed a notable increase of the number of different glycopeptides detected including glycopeptides derived from proteins never suspected of being glycosylated until then. Taken together with the intracellular transient overexpression of the PMTub gene transcription detected in infected macrophages, these results strongly suggest that the mannoprotein repertoire may undergo an adaptive regulation into the host cell. At the light of the reported PMTub overexpression in the highly pathogenic W. Beijing strain [53], one can readily suspect that during the infection, PMTub may mannosylate some specific, but still unidentified secreted proteins able to alter the host-cell microbicide response and to contribute to Mtb intracellular survival and multiplication.

In conclusion, although certainly not exhaustive, the present inventory based on MS evidence of glycosylation of the Mtb mannoproteins opens new important questionings and constitutes an essential starting point to uncover the complexity of the multiple roles and contributions of the protein-*O-*mannosylation in the Mtb virulence. In addition, even though the contribution of mannosyl residues to the activities and functionalities of the Mtb mannoproteins is still poorly understood and needs to be explored on a case-by-case basis [40] (protection against proteolysis? folding? Addressing?), the essentialness of this widespread PTM for Mtb virulence makes this process an ideal alternative target for the development of antagonists of high therapeutic potential. Indeed, the Mtb PMTub presents no overlapping with any of the current anti-tuberculous drug targets and its chemical inhibition would deprive Mtb of several potential virulence factors while limiting the emergence of compensatory resistances by spreading the selective pressure over the multiple essential mannoproteins contributing to Mtb pathogenicity.

## Figures and Tables

**Table 1:** Identification of the known mannoproteins deriving glycopeptides detected in the Mtb^WT^ culture filtrate (CF). (the putative *O-*glycosylation sites with the highest occupancy probability according to NetOGlyc 4.0 [42] predictions are indicated by underscored Ser or Thr residues; exp-mr; experimental mass; mr-delta: mass difference between the experimentally determined and the calculated masses; start-end: number of the glycopeptide starting and final amino acids in the protein sequence; var-mod: variable modification of the peptide; Hex: hexose; Ox: methionine oxidation; Ca: cysteine carbamidomethylation; Pr: cysteine propionamidation).

Gene/Protein Description	Exp_Mr	Mr_Delta	Start-End	Score	Pep_Seq	Var_Mod	Ref.
RV0175 MCE-associated membrane protein	5175.19	−0.023	100–144	16.97	DCVAATQAPDAGAMSASMQKIIECGTGDFGAQASLYTSMLVEAYQ	3 Hex, Ox, Pr	[12]
	3311.31	0.006	100–124	12.85	DCVAATQAPDAGAMSASMQKIIECG	5 Hex, 2 Ox	
Rv0432 superoxide dismutase [Cu-Zn]	3480.57	0.009	53–83	34.27	SPAPSGLSGHDEESPGAQSLTSTLTAPDGTK	3 Hex	[9]
	3134.43	0.007	55–83	15.87	APSGLSGHDEESPGAQSLTSTLTAPDGTK	2 Hex	
Rv1270c lipoprotein lprA	2356.01	0.004	34–51	17.75	ASDTAATASNGDAAMLLK	4 Hex	[12]
	2193.97	0.008	34–51	53.89	ASDTAATASNGDAAMLLK	3 Hex	
	2047.91	0.006	34–51	82.16	ASDTAATASNGDAAMLLK	2 Hex, Ox	
	2031.91	0.007	34–51	75.72	ASDTAATASNGDAAMLLK	2 Hex	
	1885.85	0.006	34–51	79.85	ASDTAATASNGDAAMLLK	1 Hex, Ox	
	1869.86	0.005	34–51	80.64	ASDTAATASNGDAAMLLK	1 Hex	
RV1368 lipoprotein lprF	3110.46	0.010	42–67	40.23	KPTTASSPSPGSPSPEAQQILQDSSK	3 Hex	[11]
	2948.41	0.007	42–67	45.9	KPTTASSPSPGSPSPEAQQILQDSSK	2 Hex	
	2786.35	0.008	42–67	41.05	KPTTASSPSPGSPSPEAQQILQDSSK	1 Hex	
	2359.11	0.003	46–67	11.61	ASSPSPGSPSPEAQQILQDSSK	1 Hex	
RV1860 alanine and proline-rich secreted protein apa	4563.39	0.012	278–321	22.07	ALAESIRPLVAPPPAPAPAPAEPAPAPAPAGEVAPTPTTPTPQR	2 Hex	[26]
	4401.33	0.010	278–321	29.05	ALAESIRPLVAPPPAPAPAPAEPAPAPAPAGEVAPTPTTPTPQR	1 Hex	
	2263.07	−0.012	309–325	20.43	EVAPTPTTPTPQRTLPA	3 Hex	
RV2068c beta-lactamase	2747.30	−0.017	25–47	17	ARHASGARPASTTLPAGADLADR	3 Hex	[12]
	2005.98	0.000	31–47	27.16	ARPASTTLPAGADLADR	2 Hex	
	1934.94	0.001	32–47	28.64	RPASTTLPAGADLADR	2 Hex	
	1778.85	0.005	33–47	20.07	PASTTLPAGADLADR	2 Hex	
	1772.81	0.004	35–47	16.3	STTLPAGADLADR	3 Hex	
	1610.75	0.000	35–47	19.21	STTLPAGADLADR	2 Hex	
	1523.72	0.001	36–47	20.21	TTLPAGADLADR	2 Hex	
RV2290 hypothetical protein	4998.26	−0.006	47–85	13.09	ATVMVEGHTHTISGVVECRTSPAVRTATPSESGTQTTR	6 Hex	[12]
	1497.68	0.003	73–85	23.23	TATPSESGTQTTR	1 Hex	
RV2799 hypothetical protein	2096.95	0.003	68–83	26.33	SPIVATTDPSPFDPCR	2 Hex, Pr	[12]
	2082.94	0.006	68–83	61.05	SPIVATTDPSPFDPCR	2 Hex, Ca	
	1920.88	0.002	68–83	37.81	SPIVATTDPSPFDPCR	1 Hex, Ca	
RV2873 cell surface lipoprotein MPB83	7063.14	−0.022	44–106	11.82	AAPVTTAAMADPAADLIGRGCAQYAAQNPTGPGSVAGMAQDPVATAASNNPMLSTLTSALSGK	6 Hex, 2Ox	[8]
	4982.32	0.012	21–62	10.69	FLAGCSSTKPVSQDTSPKPATSPAAPVTTAAMADPAADLIGR	5 Hex, Ox	
	1974.89	0.003	48–62	14.46	TTAAMADPAADLIGR	3 Hex, Ox	
	1958.90	0.011	48–62	43.02	TTAAMADPAADLIGR	3 Hex	
RV3491 hypothetical protein	2022.08	0.005	164–180	25.55	QPFSLQLIGPPPSPVQR	1 Hex	[12]
	1649.91	0.007	167–180	36.78	SLQLIGPPPSPVQR	1 Hex	
RV3835 hypothetical protein	6066.10	−0.001	362–415	20.03	LNLPPIPLQLPTPRPAPPAQQLPSTPPGTQHLPAQQPVVTPTRPPESHAPASAA	3 Hex	[12]
	1730.00	−0.001	362–375	10.6	LNLPPIPLQLPTPR	1 Hex	

**Table 2:** Identification of the novel glycopeptide detected in the Mtb^WT^ CF by the improved “neutral loss” post analytical MS^2^ data processing. (* and ^§^: See Figure 1 and Figure S3 for detailed identification; for additional legend see Table 1).

Gene / Protein Description	Exp_Mr	Mr_Delta	Start-End	Score	Sequence (Predicted Glycosylation Sites)	Var_Mod	#MS^2^
**RV0315** **hypothetical protein**	3615.74	0.007	38–74	47.14	APAGPTPAPAAPAAATGGLLFHDEFDGPAGSVPDPSK ^§^	1 Hex	11
	3453.68	−0.001	38–74	46.3	APAGPTPAPAAPAAATGGLLFHDEFDGPAGSVPDPSK ^§^		(1)
	3544.70	0.005	39–74	28.62	PAGPTPAPAAPAAATGGLLFHDEFDGPAGSVPDPSK	1 Hex	7
	3382.64	−0.010	39–74	36.27	PAGPTPAPAAPAAATGGLLFHDEFDGPAGSVPDPSK		(1)
**RV0838** **D-alanyl-D-alanine dipeptidase**	2051.97	0.007	47–62	11.36	TTTPSGPVPPVSEAAR *	3 Hex	1
	1889.92	0.006	47–62	18.28	TTTPSGPVPPVSEAAR *	2 Hex	6
**RV1411c** **lipoprotein lprG**	1115.61	0.001	228–236	13.65	VQVTKPPVS ^§^	1 Hex	2
	953.55	−0.001	228–236	39.14	VQVTKPPVS ^§^		(12)
RV1625c adenylate cyclase	1658.73	0.010	28–37	16.01	TQARAPTQHY	3Hex, Da	2
RV1731 succinate-dehydrogenase	1632.82	0.004	2–14	26.43	PAPSAEVFDRLRN	1 Hex	4
RV1935c enoyl-CoA hydratase	1497.68	−0.001	62–73	16.82	AFELAEADDTVR	1 Hex	2
Rv2563 ABC transporter	1705.82	0.004	188–202	12.89	GQPTISSIGIDGMPR	1 Hex, Ox	3
Rv3209 Hypothetical protein	3813.80	−0.002	156–186	12.78	LYSRLNCSIVNTGAQTVVASTNNAIIATCTR	3 Hex, 3 Da, Pr	2

**Table 3:** Glycosylated peptides identified in the independent analysis of the Mtb^WT^ CF. (Newly identified sequences are in bold; *: See Figure S4 for detailed identification and Table 1 for additional legend).

Peptide Sequence	Exper. Mr	Expec. Mr	Modification	Score	Gene: Protein Description
GSGDSHNGGVTTPPLDDLVGDDLVPYR *	2915.33	2752.28	Hex, Da	19.64	Rv0237: beta-glucosidase
APAGPTPAPAAPAAATGGLLFHDEFDGPAGSVPDPSK	*3615.74*	*3453.67*	*Hex*	*27.93*	*Rv0315: hypothetical protein*
SPAPSGLSGHDEESPGAQSLTSTLTAPDGTK	*3480.57*	*2994.40*	*3 Hex*	*21.75*	*Rv0432: superoxide dismutase*
GNDDNVTGGGATTGQASAK *	2205.93	1719.76	3 Hex	47.62	Rv0928: phosphate-binding protein pstS 3
ASDTAATASNGDAAMLLK	*1885.85*	*1706.80*	*Hex, Ox, Da*	*87.28*	*Rv1270c: lipoprotein lprA*
TLSGDLTTNPTAATGNVK	*1922.94*	*1759.88*	*Hex, Da*	*12.82*	*Rv1411c: lipoprotein lprG*
ASTTLPAGADLADR	*1681.79*	*1357.67*	*2 Hex*	*19.5*	*Rv2068c: beta-lactamase*
TATPSESGTQTTR	*1497.68*	*1335.62*	*Hex*	*36.17*	*Rv2290: hypothetical protein*
GAPSTAGPCEIVPNGTPAPK	2082.98	1862.91	Hex, Ca, Da	30.21	Rv2394: gamma-glutamyltransferase
VPVSPTASDPALLAEIR	2059.06	1734.94	2 Hex	35.43	Rv2945c: lipoprotein lPpx
QPFSLQLIGPPPSPVQR	*2022.08*	*1860.02*	*Hex*	*23*	*Rv3491: hypothetical protein*
GETTTAAGTTASPGAASGPK	2380.04	1731.82	4 Hex	36.99	Rv3763: lipoprotein lpqH
LNLPPIPLQLPTPR	*1730.00*	*1567.93*	*Hex*	*33.46*	*Rv3835: hypothetical protein*

**Table 4:** Peptides glycoforms detected in cell–lysate protein extracts of the Mtb^WT^ and PMTub-overexpressing strain. Specific cell-associated glycosylated proteins are noted in bold and their occurrence in the Mtb^WT^ and /or in the complemented strain overexpressing the PMTub gene (Cp) are reported in the right column. (see Table 1 for additional legend).

Gene	Protein Description	exp_mr	mr_Delta (ppm)	Start-End	Score	Peptide Sequence	Var_Mod	Wt/Cp
**RV0199**	**hypothetical protein**	1833.94	4.39	151–166	28.45	(R)ELDTQSGAARPVVTTK(L)	Hex	Wt/Cp
		1671.88	2.09	151–166	54.66	(R)ELDTQSGAARPVVTTK(L)		Wt/Cp
**RV0227c**	**hypothetical protein**	1667.84	0.02	138–151	28.6	(R)GLNDENPPTAIPLR(H)	Hex	Wt/Cp
		1505.78	0.57	138–151	49.36	(R)GLNDENPPTAIPLR(H)		Wt/Cp
RV0432	superoxide dismutase [Cu-Zn]	2966.33	−1.00	57–83	30.15	(P)SGLSGHDEESPGAQSLTSTLTAPDGTK(V)	2 Hex	Wt/Cp
		3480.56	0.06	53–83	18.28	(G)SPAPSGLSGHDEESPGAQSLTSTLTAPDGTK(V)	3 Hex	Wt/Cp
**RV1223**	**htrA**	1662.80	−2.39	214–228	50.29	(K)VTLSTTGNAQEPAGR(F)	Hex	-/Cp
		1501.74	0.11	214–228	76.54	(K)VTLSTTGNAQEPAGR(F)	Da	-/Cp
RV1270c	lipoprotein lprA	2356.01	−0.18	34–51	75.21	(K)ASDTAATASNGDAAMLLK(Q)	4 Hex, Da	Wt/Cp
		2193.96	1.41	34–51	66.26	(K)ASDTAATASNGDAAMLLK(Q)	3 Hex, Da	-/Cp
		2030.93	2.89	34–51	79.32	(K)ASDTAATASNGDAAMLLK(Q)	2 Hex	Wt/Cp
		1869.85	−0.08	34–51	62.35	(K)ASDTAATASNGDAAMLLK(Q)	Hex, Da	Wt/Cp
		1868.87	3.90	34–51	67.93	(K)ASDTAATASNGDAAMLLK(Q)	Hex	Wt/Cp
		1707.80	−0.75	34–51	85.51	(K)ASDTAATASNGDAAMLLK(Q)	Da	Wt/Cp
RV1368	lipoprotein lprF	3272.50	−0.54	42–67	43.73	(K)KPTTASSPSPGSPSPEAQQILQDSSK(A)	4 Hex	Wt/Cp
		3110.45	−0.19	42–67	47.88	(K)KPTTASSPSPGSPSPEAQQILQDSSK(A)	3 Hex	Wt/Cp
		2948.39	−2.28	42–67	61.82	(K)KPTTASSPSPGSPSPEAQQILQDSSK(A)	2 Hex	Wt/Cp
		2786.34	−0.74	42–67	89.33	(K)KPTTASSPSPGSPSPEAQQILQDSSK(A)	Hex	Wt/Cp
		2624.29	−0.54	42–67	72.17	(K)KPTTASSPSPGSPSPEAQQILQDSSK(A)		Wt/Cp
RV1411c	lipoprotein lprG	2084.00	−1.07	75–92	67.17	(K)TLSGDLTTNPTAATGNVK(L)	2 Hex	-/Cp
		1921.95	0.15	75–92	96.46	(K)TLSGDLTTNPTAATGNVK(L)	Hex	Wt/Cp
		1759.89	−0.41	75–92	98.98	(K)TLSGDLTTNPTAATGNVK(L)		Wt/Cp
		1277.66	−0.19	228–236	11.3	(K)VQVTKPPVS(-)	2 Hex	Wt/-
		1115.61	−1.23	228–236	24.38	(K)VQVTKPPVS(-)	Hex	Wt/Cp
		953.55	−2.24	228–236	40.29	(K)VQVTKPPVS(-)		Wt/Cp
**RV1677**	**hypothetical protein dsbF**	3563.73	−1.49	26–57	11	(K)SQPAVAPTGDAAAATQVPAGQTVPAQLQFSAK(T)	3 Hex	Wt/Cp
		3401.68	−0.35	26–57	32.98	(K)SQPAVAPTGDAAAATQVPAGQTVPAQLQFSAK(T)	2 Hex	Wt/Cp
		3239.63	0.45	26–57	49.38	(K)SQPAVAPTGDAAAATQVPAGQTVPAQLQFSAK(T)	Hex	-/Cp
		3077.58	0.03	26–57	42.18	(K)SQPAVAPTGDAAAATQVPAGQTVPAQLQFSAK(T)		Wt/Cp
**RV1743**	**serine/threonine-protein kinase pknE**	1937.95	−0.11	369–384	21.41	(R)TSAPPPPPPADAVELR(V)	2 Hex	Wt/-
		1613.84	−2.36	369–384	22.97	(R)TSAPPPPPPADAVELR(V)		Wt/Cp
**RV1748**	**hypothetical protein**	2889.32	−1.73	55–81	30.26	(R)LGEASGDLASDSPAMPRPTTVENDAAR(W)	Hex	Wt/-
		2727.28	0.69	55–81	69.44	(R)LGEASGDLASDSPAMPRPTTVENDAAR(W)		Wt/Cp
RV2068c	beta-lactamase	1778.84	0.05	33–47	13.4	(R)PASTTLPAGADLADR(F)	2 Hex	-/Cp
		1454.74	0.11	33–47	61.69	(R)PASTTLPAGADLADR(F)		-/Cp
**RV2744c**	**phage shock protein A**	1459.71	−1.44	240–253	52.1	(R)GEALPAGGTTATPR(P)	Hex	Wt/Cp
		1297.66	−0.89	240–253	88.98	(R)GEALPAGGTTATPR(P)		Wt/Cp
RV2799	hypothetical protein	2082.93	2.19	68–83	43.65	(K)SPIVATTDPSPFDPCR(D)	2 Hex, Ca	Wt/Cp
**RV2969c**	**hypothetical protein**	2076.05	−0.45	236–255	56.32	(K)IKEIVGDVPGIDSAAATATS(-)	Hex	Wt/-
		1913.99	−0.25	236–255	64.95	(K)IKEIVGDVPGIDSAAATATS(-)		Wt/Cp
		1851.97	−0.65	202–218	39.2	(K)VDGLAAAVNVHATPTVR(V)	Hex	Wt/-
		1689.91	−1.01	202–218	82.14	(K)VDGLAAAVNVHATPTVR(V)		Wt/-
**RV3090**	**hypothetical protein**	2354.19	0.16	274–295	44.23	(K)GISPLGCWPGSSALPTIAVPGR(-)	Hex, Ca	Wt/Cp
		2192.14	0.77	274–295	79.41	(K)GISPLGCWPGSSALPTIAVPGR(-)	Ca	Wt/Cp
RV3491	hypothetical protein	2022.08	−0.37	164–180	38.76	(K)QPFSLQLIGPPPSPVQR(Y)	Hex	-/Cp
		1649.90	1.07	167–180	19.71	(F)SLQLIGPPPSPVQR(Y)	Hex	Wt/Cp
		1487.84	−3.18	167–180	32.07	(F)SLQLIGPPPSPVQR(Y)		Wt/-
**RV3584**	**lipoprotein lpqE**	1998.96	2.22	164–182	47.69	(K)AGQGSVMVPISAGLATPHE(-)	Hex, Ox	-/Cp
		1982.96	0.11	164–182	56.18	(K)AGQGSVMVPISAGLATPHE(-)	Hex	Wt/-
		1820.91	−0.63	164–182	110.3	(K)AGQGSVMVPISAGLATPHE(-)		Wt/Cp
**RV3623**	**hypothetical protein**	2219.11	−0.33	195–215	64.2	(K)VISISEASGAAPTHEAPAPPR(G)	Hex	Wt/Cp
		2057.06	3.52	195–215	91.34	(K)VISISEASGAAPTHEAPAPPR(G)		Wt/Cp
		2454.19	0.84	99–120	28.12	(R)VTVAPQYSNPEPAGTATITGYR(A)	Hex	Wt/-
		2292.14	−0.79	99–120	93.23	(R)VTVAPQYSNPEPAGTATITGYR(A)		Wt/-
RV3763	lipoprotein lpqH	3623.49	1.18	27–51	17.12	(K)STTGSGETTTAAGTTASPGAASGPK(V)	9 Hex	Wt/Cp
		3461.43	0.06	27–51	32.84	(K)STTGSGETTTAAGTTASPGAASGPK(V)	8 Hex	Wt/Cp
		3299.38	1.35	27–51	38.84	(K)STTGSGETTTAAGTTASPGAASGPK(V)	7 Hex	Wt/Cp
		3137.33	−0.11	27–51	19.27	(K)STTGSGETTTAAGTTASPGAASGPK(V)	6 Hex	Wt/Cp
		2813.22	0.64	27–51	31.75	(K)STTGSGETTTAAGTTASPGAASGPK(V)	4 Hex	Wt/Cp
		2651.17	1.27	27–51	51.82	(K)STTGSGETTTAAGTTASPGAASGPK(V)	3 Hex	Wt/Cp
		2489.12	1.19	27–51	69.7	(K)STTGSGETTTAAGTTASPGAASGPK(V)	2 Hex	Wt/Cp
		2327.06	1.42	27–51	72.66	(K)STTGSGETTTAAGTTASPGAASGPK(V)	Hex	-/Cp
		2165.00	−1.59	27–51	111.1	(K)STTGSGETTTAAGTTASPGAASGPK(V)		Wt/Cp
RV3835	hypothetical protein	2030.98	0.87	72–87	13.38	(R)LAGYIASNPVPSTGAK(I)	3 Hex	-/Cp
		1706.87	0.35	72–87	26.07	(R)LAGYIASNPVPSTGAK(I)	Hex	-/Cp
**RV3869**	**ESX-1 secretion system protein eccB1**	1978.95	0.12	454–470	43.68	(K)DAALLEHDTLPADPSPR(K)	Hex	Wt/Cp
		1816.89	−1.10	454–470	60.59	(K)DAALLEHDTLPADPSPR(K)		Wt/Cp

**Table 5:** New glycosylated peptides detected in the secretome of the Mtb complemented strain overexpressing the PMTub gene. (Hex-num: hexose number, for additional legend see Table 1).

Gene	prot_desc	pep_exp_mr	start-pep_end	pep_score	pep_seq	pep_var_mod
RV0822c	hypothetical protein	1637.79	522-534	16.01	LGNTPSTPPTTTK	2 Hex
		1475.74	522-534	35.88	LGNTPSTPPTTTK	Hex
		1313.68	522-534	32.55	LGNTPSTPPTTTK	
RV1157c	hypothetical protein	1294.64	211-224	23.21	AAAPAPASAAPAPA	Hex
		1132.59	211-224	22.01	AAAPAPASAAPAPA	
		1365.68	211-225	10.82	AAAPAPASAAPAPAA	Hex
RV2094c	TatA/E family twin arginine-targeting protein translocase	1503.74	55-66	22.44	SIETPTPVQSQR	Hex
		1341.69	55-66	52.68	SIETPTPVQSQR	
RV2220	glutamine synthetase 1	1340.63	2-11	21.43	TEKTPDDVFK	Hex
		1178.58	2-11	55.91	TEKTPDDVFK	
RV3244c	lipoprotein lpqB	3156.58	29-53	30.03	GTVERPVPSNLPKPSPGMDPDVLLR	3 Hex
		2994.52	29-53	22.99	GTVERPVPSNLPKPSPGMDPDVLLR	2 Hex
RV3413c	hypothetical protein	1646.78	285-299	30.05	PAGQPAPETPVSPTH	Hex
		1484.73	285-299	50.33	PAGQPAPETPVSPTH	
RV3705A	hypothetical protein	1860.89	115-129	15.18	VGPTGPGPTTAPARP	3 Hex
		1698.83	115-129	30.6	VGPTGPGPTTAPARP	2 Hex
RV3706c	hypothetical protein	2981.43	81-106	8.08	AVRPGPGPGGPGQVPSSVSPPATPAP	4 Hex
		2819.37	81-106	25.94	AVRPGPGPGGPGQVPSSVSPPATPAP	3 Hex
		2657.32	81-106	26.69	AVRPGPGPGGPGQVPSSVSPPATPAP	2 Hex
		2910.39	82-106	16.99	VRPGPGPGGPGQVPSSVSPPATPAP	4 Hex
		2748.34	82-106	20.4	VRPGPGPGGPGQVPSSVSPPATPAP	3 Hex
		2586.28	82-106	25.37	VRPGPGPGGPGQVPSSVSPPATPAP	2 Hex

## Supplementary Materials

The following are available online, Supplemental Figures to Tonini et al.docx: Supplemental Figures S1–S5, Table S1: List of the proteins detected in the culture filtrate of the Mtb H37Rv, Table S2: List of the 223 Mtb culture filtrate putative glycopeptides, Table S3: List of the putative glycopeptides detected in the culture filtrate of the Mtb Rv1002c KO Mutant, Table S4: Lists of the validated proteins and glycopeptides detected in the culture filtrate of the Mtb strain overexpressing the PMTub, Table S5: Relative expression of Rv1002c in the Mtb strain overexpressing the PMTub gene, Table S6: qPCR Raw data of the relative expression of the PMT Rv1002c gene during infection of MH-S macrophages.
