# Supplementary material for: Potential Plasticity of the Mannoprotein Repertoire Associated to Mycobacterium tuberculosis Virulence Unveiled by Mass Spectrometry-Based Glycoproteomics
Source: Molecules. 2020 May 18;25(10):2348. doi: 10.3390/molecules25102348 (PMC7287972; doi:10.3390/molecules25102348)
Supplement: Supplementary file 1 [file molecules-25-02348-s001.zip › Supplemental Figures to Tonini et al.doc]

**Supplemental figures S1 to S5 : “*Potential Plasticity of the Mannoprotein Repertoire Associated to Mycobacterium tuberculosis Virulence”*** by L. Tonini *et al*.

**Figure S1**: Scheme depicting the basic steps of the SDS-PAGE based MS shotgun glycoproteomic and post-analytical data processing workflow for the high confidence identification of *M. tuberculosis* (Mtb H37Rv) glycopeptides.

A) Logical diagram pointing out the quantitative improvement provided by the neutral loss signature search strategy (right panel) compared to a “conventional” post analytical MS2 data processing (left panel).

To increase the Mtb proteome coverage, the protein extract is sub-fractionated by SDS-PAGE in 17 bands that are individually submitted to LTQ-Orbitrap instrument LC-MS/MS proteomic analysis. The typical workflow for protein identification figured on the left panel illustrates the Mascot 2.2 search engine conventional built-in filters (symbolized by grey trapeziums), applied for glycoprotein identification (glycopeptides (G-pept) or glycoproteins (G-prot) indicated numbers are given by way of an example and correspond to those of the analysis of the Mtb CF protein extract Mtb-C1 (see Figure 3); “PTM=Hex”: post translational O-glycosyl modified protein characterized by a mass difference of n x162uma (n=1 to 3) corresponding to the neutral loss of hexose residues from substituted serine or threonine of the glycopeptide precursor ion; FDR: false discovery rate.

Right panel: the screening of the fragmentation spectra displaying a consistent “Neutral Loss Signature” (defined as the presence among the 30 most intense peaks, of fragment ions with a relative intensity to the major ion of ≥ 10% and a mass fitting with the loss of hexose residues as neutral fragments), either prior or after the MS data processing by the search engine, drastically tightens the number of glycopeptides potential spectra that are further subjected to manual curation.

B) Final logical decisional tree used for the high confidence de novo identification of the Mtb glycopeptides (see main text for description).

A)

B)

**Figure S2:** : Label free quantification of the proteins detected in the culture filtrate protein extracts of the wild type Mtb strain (WT) and the ΔRv1002c PMT deleted mutant (MT) assessing the absence of effect of the PMT invalidation on the glycoproteins abundances. 100 μg of culture filtrate protein extract from 3 biological replicates of the wild type Mtb strain and the *rv1002c^pBlaf*^* complemented mutant grown for 4 weeks in Sauton broth were concentrated in 4.4% stacking polyacrylamide gels and processed as described in the Material & Method section. After tryptic in-gel digestion, the resulting peptides were analyzed by nanoLC-MS/MS. MS data of the three independent experiments were combined and analyzed by the MaxQuant label-free quantitation algorithm as previously described . A) Volcano plot of the relative protein abundances (CP *vs* WT) highlighting the up- and down-regulated proteins in the complemented mutant. B) Normalized intensities of the putatively glycosylated Mtb proteines detected in the culture filtrates of the *rv1002c^pBlaf*^* complemented mutant (Mean LFQ Sauton P2SCP) and of the parent Mtb H37Rv wild type strain (Mean LFQ Sauton P2SWT).

**A)**

B)

**Figure S3**: Evidences of glycosylation of novel Mtb mannoproteins: chromatographic and mass spectrometric evidences of glycosylation of the Rv0315 P38-74 peptide detected in the Mtb H37Rv secretome. See Table 2.

m/z=1152.23 ±10ppm

m/z=1206.25 ±10ppm

**Figure S-3 (**continued)**:** chromatographic and mass spectrometric evidences of glycosylation of the Rv0315 P39-74 peptide detected in the Mtb H37Rv secretome. See Table 2.

m/z=1128.53 ±10ppm

m/z=1182.57 ±10ppm

**Figure S-3 (**continued)**:** chromatographic and mass spectrometric evidences of glycosylation of the Rv1411c (LprG lipoprotein) P228-236 peptide detected in the Mtb H37Rv secretome. See Table 2.

m/z=477.78 ±20ppm

m/z=558.81 ±20ppm

**Figure S4**: Evidences of glycosylation of novel Mtb mannoproteins (continued): chromatographic and mass spectrometric evidences of glycosylation of the Rv0237 P231-257 peptide. See Table 3.

m/z=972.784 ±2ppm

m/z=918.767 ±2ppm

[M+3H]^3+^= 918.767

[M+3H]^3+^= 972.784

[M-162+3H]^3+^

**Figure S-4** (continued): chromatographic and mass spectrometric evidences of glycosylation of the Rv0928 P24-42 peptide. See Table 3.

m/z=1103.97

m/z=1022.944

m/z=860.891

±5ppm

[M+2H]^2+^= 1103.97

[M-486+2H]^2+^

[M-324+2H]^2+^

[M-162+2H]^2+^

[M-324+2H]^2+^

[M-162+2H]^2+^

[M+2H]^2+^= 860.89

**Figure S5**: Label free quantification of the proteins detected in the culture filtrate protein extracts of the *rv1002c^pBlaf*^* complemented mutant (CP) and the wild type Mtb strain (WT) assessing the absence of effect of the PMT over-expression on the glycoproteins abundances. 100 μg of culture filtrate protein extract from 3 biological replicates of the wild type Mtb strain and the *rv1002c^pBlaf*^* complemented mutant grown for 4 weeks in Sauton broth were concentrated in 4.4% stacking polyacrylamide gels and processed as described in the Material & Method section. After tryptic in-gel digestion, the resulting peptides were analyzed by nanoLC-MS/MS. MS data of the three independent experiments were combined and analyzed by the MaxQuant label-free quantitation algorithm as previously described . A) Volcano plot of the relative protein abundances (CP *vs* WT) highlighting the up- and down-regulated proteins in the complemented mutant. B) Normalized intensities of the putatively glycosylated Mtb proteines detected in the culture filtrates of the *rv1002c^pBlaf*^* complemented mutant (Mean LFQ Sauton P2SCP) and of the parent Mtb H37Rv wild type strain (Mean LFQ Sauton P2SWT).

A)

B)
